# Promoting community readiness for physical activity among older adults in Germany – protocol of the ready to change intervention trial

**DOI:** 10.1186/s12889-016-2761-2

**Published:** 2016-02-01

**Authors:** Tilman Brand, Dirk Gansefort, Heinz Rothgang, Sabine Röseler, Jochen Meyer, Hajo Zeeb

**Affiliations:** 1 Department Prevention and Evaluation, Leibniz Institute for Prevention Research and Epidemiology – BIPS, Achterstr. 30, 28359 Bremen, Germany; 2 University of Bremen, Centre for Social Policy Research (ZeS), Mary-Somerville-Straße 3, 28359 Bremen, Germany; 3 Gesundheitswirtschaft Nordwest e.V., Hinter dem Schütting 8, 28195 Bremen, Germany; 4 OFFIS Institute for Information Technology, R&D Division Health, Escherweg 2, 26121 Oldenburg, Germany; 5 Health Sciences Bremen, University of Bremen, Bibliothekstraße 1, 28359 Bremen, Germany

**Keywords:** Physical activity, Older adults, Elderly, Primary prevention, Health equity, Social determinants

## Abstract

**Background:**

Healthy ageing is an important concern for many societies facing the challenge of an ageing population. Physical activity (PA) is a major contributor to healthy ageing; however insufficient PA levels are prevalent in old age in Germany. Community capacity building and community involvement are often recommended as key strategies to improve equitable access to prevention and health promotion. However, evidence for the effectiveness of these strategies is scarce. This study aims to assess the community readiness for PA promotion in local environments and to analyse the utility of strategies to increase community readiness for reaching vulnerable groups.

**Methods/Design:**

We designed a mixed method intervention trial comprising three study modules. The first module includes an assessment of community readiness for PA interventions in older adults. The assessment is carried out in a sample of 24 municipalities in the Northwest of Germany using structured key informant interviews. In the second module, eight municipalities with the low community readiness are selected from the sample and randomly assigned to one of two study groups: active enhancement of community readiness (intervention) versus no enhancement (control). After enhancing community readiness in the active enhancement group, older adults in both study groups will be recruited for participation in a PA intervention. Participation rates are compared between the study groups to evaluate the effects of the intervention. In addition, a cost-effectiveness analysis is carried out calculating recruitment costs per person reached in the two study groups. In the third module, qualitative interviews are conducted with participants and non-participants of the PA intervention exploring reasons for participation or non-participation.

**Discussion:**

This study offers the potential to contribute to the evidence base of reaching vulnerable older adults for PA interventions and provide ideas on how to reduce participation barriers. Its findings will inform governmental authorities, professionals, academics, and NGOs with an estimate of resources necessary to achieve equitable access to physical activity programs for vulnerable older adults.

**Trial registration:**

German Clinical Trials Register DRKS00009564 (Date of registration 03-11-2015)

## Background

Healthy ageing is an important concern for many societies facing the challenge of an ageing population. Physical activity (PA) is a major contributor to healthy ageing [1, 2] that has been shown to reduce risks for many chronic diseases and injuries. Enhanced PA improves cardiometabolic markers, lowers resting blood pressure and decreases the risk for diabetes mellitus type II [3–5]. Positive mental health and cognitive effects of exercise have also been described [6]. The World Health Organization (WHO) recommends that older adults aged 65 years and above should engage in at least 150 min of moderate-intensity aerobic physical activity weekly in order to gain and/or maintain health benefits [7]. Flexibility and strength training is also recommended at least two times per week to increase mobility skills and reduce the risk of falling [8]. Thus, PA is a key health resource for an ageing community. In spite of these well-known beneficial effects, only one third of older adults in Germany are physically active; 25.4% of male and 15.5% of female persons over the age of 70 reach the WHO-recommended levels [9]. In addition, there are notable differences in participation rates in behaviour-oriented health promotion programs across socioeconomic status (SES) and sex [10]. While a wide range of primary prevention activities on PA are available in Germany [11], a major knowledge gap in identifying which interventions are successful for specific target populations remains. Evidence suggests that tailoring interventions to the needs of the target group increases the intervention’s reach and efficacy [12]. Furthermore, it has been demonstrated that tailoring PA interventions to stages of individual behaviour change may enhance intervention reach and effectiveness [13]. This may also be the case at a community level. A systematic review by Stith et al. [14] concluded that a community should fulfil four conditions before a preventive intervention can be successfully implemented: (i) sufficient community capacity exists, i.e., a functioning community coalition is installed; (ii) the community recognizes that there is a problem, and that existing programs cannot solve the problem sufficiently (iii) a key person/ organization is identified; and (iv) an appropriate climate for implementation exists, i.e., stakeholders benefit from participation or at least have no drawbacks from or high cost for participation. The use of community capacity building approaches to reach and engage at-risk groups and local stakeholders in their natural living environment is a promising way of avoiding social selectiveness in service participation and achieving sustainable program implementation [15–17]. The concept of community readiness outlines an approach to increase a community’s readiness to participate in a health behaviour change intervention. It applies a stage-based behaviour change model to the community level [18, 19]. According to this concept, a certain degree of problem awareness and pre-planning in the community is crucial for a health promotion intervention to be successfully implemented [18–20]. It is therefore recommended to assess and, if necessary, increase community readiness before starting an intervention. Depending on the stage of community readiness (9 stages ranging from no problem awareness to professionalization of interventions), the model suggests different strategies to enhance program implementation. The model has already been successfully applied in diverse fields of community based health promotion such as HIV/AIDS prevention, suicide prevention and prevention of cardiovascular disease [21–23]. These studies report on the usefulness of the model for community capacity building. However its utility for reaching vulnerable populations for PA interventions and overall cost-effectiveness have not yet been systematically investigated.

The *Ready To Change* (RTC) study is part of the *Physical Activity And Health Equity: Primary Prevention For Healthy Ageing* (AEQUIPA) project, a regional prevention research network in Northwest Germany funded by the German Federal Ministry of Education and Research (BMBF). The AEQUIPA research network includes several interlinked projects which employ theory-based empirical research methods to develop, implement and evaluate PA and mobility interventions for older adults aged 65–75 years. The network’s overall aim is to strengthen the evidence base for PA in the context of healthy ageing. The RTC study investigates the utility of strategies to increase community readiness for older adults’ participation in a PA intervention. As reaching vulnerable groups is among the aims of community based strategies for health promotion, we specifically investigate whether increasing community readiness leads to higher participation rates in traditionally hard-to-reach population groups (e.g., low SES, migrants, physically inactive or obese persons). Models and empirical investigations of access to health service indicate that service uptake is influenced by system factors, such as community readiness, but also by individual factors (e.g., attitude, knowledge, need) [24–26]. Hence, individual factors have to be considered when analysing non-participation in PA interventions. A qualitative investigation may add to a deeper understanding of non-participation by providing a biographical account of motives that led to non-participation. Thus, an exploration of reasons for (non-)participation was added to the aims of this study.

In summary, the RTC study aims to:

- Assess the community readiness for older adults’ PA participation in selected municipalities.
- Investigate the utility of strategies to increase community readiness for engaging vulnerable older adults in a PA intervention.
- Examine reasons for non-participation in existing PA interventions among older adults.

## Methods/Design

We designed a mixed methods intervention trial including three study modules. The study is located in the Bremen-Oldenburg metropolitan region. The region is a geographically and politically defined area in the Northwest of Germany comprising about 150 rural and urban municipalities. The study started in February 2015 and will last until January 2018.

### Community readiness assessment

In the first study module, a cross-sectional community readiness assessment for PA interventions in older adults (65–75 years) is conducted in a sample of municipalities (settlements and districts) within the Bremen-Oldenburg metropolitan region. Municipalities are selected if they already display a comparably high proportion of older adults or if they expect a high increase in the proportion of older adults over the next decade. Overall, 24 municipalities (12 rural, 12 urban) are included in this assessment.

The community readiness assessment is based on a structured interview administered to key informants in each of the selected communities. Key informants are, for example, representatives from local public authorities, senior citizen organisations, sports clubs, for-profit PA providers, faith-based organisations, and members of the target group (i.e., older adults living in the municipality). Strategies to identify key informant include internet searches and snowball sampling via interviewees. The interview comprises 36 semi-structured questions covering the five key dimension of community readiness: (1) community knowledge of the issue, (2) community knowledge of the efforts, (3) community climate, (4) leadership, and (5) resources [20]. In each municipality five key informant interviews are conducted [18]. Interviews are administered either face-to-face or as telephone interviews. Transcripts of the responses are analysed by two independent raters and scored from 1 to 9 for each readiness dimension, according to rating criteria described in the community readiness assessment manual [20]. The raters agree upon a final score for each municipality.

### Effects of strategies to increase community readiness

**Fig. 1:**
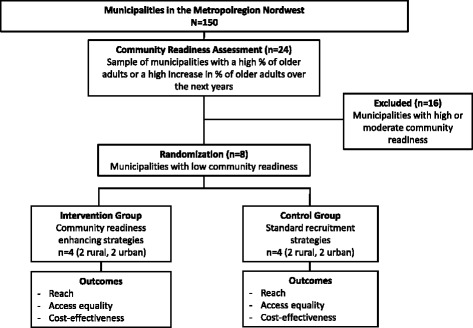
Design of the Ready To Change intervention trial

In the second study module, eight municipalities identified as having the lowest community readiness score in module 1 are randomly assigned to one of two study groups: active enhancement of community readiness (intervention) versus no enhancement (control; see Fig. 1).

#### Treatment conditions

In the intervention group strategies are applied to increase community readiness for PA interventions in older adults. The community readiness concept suggests selecting strategies according to the stage of readiness a community has attained. Practical strategies for the lower readiness stages include (a) the identification of local key stakeholders via one-on-one visits with community leaders, (b) information campaigns to increase problem awareness in the community (articles to local newsletters, presentation of in-depth local statistics), (c) the establishment of a community working group on PA in older adults, and (d) the recruitment of older adults for the PA intervention via local health practitioners [19]. Intervention materials are developed to support the information campaign and the installation of the working group. Furthermore, technology-based interactive tools are provided to engage stakeholders and citizens in PA, such as public displays to visualize a group’s activity, or an online social network for uploading and commenting on photos of places and facilities in the community where one can be physically active.

In the control group no strategies are applied to increase community readiness.

After setting up the local working group in the intervention group, older adults aged 65 to 75 years are recruited for participation in a PA intervention in both study groups. The PA intervention is based on regular group meetings and a simple counselling aid to facilitate self-regulation of PA behaviour. The recruitment process comprises standard recruitment strategies that are applied in both study groups, such as newspaper articles, public service advertising, and direct mailing obtained from the local residents’ registration offices. In addition to the standard procedures, the built-up local networks in the intervention communities are involved in the recruitment process using their personal or organisational capacities to reach older adults for the intervention.

#### Outcomes

Reach of the PA intervention is chosen as a main outcome criterion for evaluating the effects of strategies to enhance community readiness. According to the RE-AIM model (Reach Efficacy Adoption Implementation Maintenance), programme reach is defined as the number of program participants divided by the number of the target population in the municipalities [27]. Administrative data is used to determine the size of the target population for the denominator. Apart from reach, access equality is evaluated as another outcome criterion. We define access equality as number and proportion of vulnerable older adults among the participants of the PA Intervention. Vulnerability is defined as having at least one socio-demographic risk factor (low socioeconomic status or migrant background or male) and one health-related risk factor (low level of physical activity or being obese). Information on the participant characteristics is gathered during baseline assessment for the PA intervention. From these variables, an index is constructed to distinguish between vulnerable and non-vulnerable older adults. Cost-effectiveness is used as the third outcome variable. The recruitment costs per person are calculated for the two study groups. Recruitment costs for the intervention group also include per capita shares of the overall costs of the community readiness assessment and the community readiness strategies described above.

#### Statistical analysis

To assess the effects of the community readiness enhancing strategies, odds ratios (OR) are calculated (reached versus non-reached in intervention and control group in a 2 × 2 table). As the size of the target population varies between the municipalities and because the control and intervention municipalities are selected at a later stage of the study, exact determination of power and sample size is not possible. However, a population size of at least 2,000 older adults aged 65 to 75 years in each of the eight municipalities would allow us to detect differences in programme reach at the level of OR = 1.5 (α = 0.05, power = 0.80), stratifying for region (rural/urban) and assuming that small proportions of the population will be reached (3% reached in the intervention group vs. 2% reached in the control group). A population size of at least 2,000 older adults is a realistic assumption as almost all of the 24 municipalities in the community readiness assessment have even more inhabitants in this age group.

For the analysis of the costs, the difference in recruitment costs is divided by the difference in effects to obtain the incremental cost-effectiveness rate (ICER). The effect is measured in terms of participation rates and the rate of participants from vulnerable groups.

### Reasons for non-participation in PA interventions

The third module focusses on biographical and cultural reasons why vulnerable older adults participate or do not participate in PA interventions, and which special recruitment strategies can be identified for underrepresented target groups. For this purpose, 10 to 15 qualitative interviews with participants and non-participants of the PA intervention are conducted (ratio 1:3). Potential interviewees are identified throughout the recruitment process in module 2. Grounded theory methodology is used for sampling and analysing the interview material [28].

### Ethics statement and consent

The study protocol was approved by the Ethics Committee of the University Bremen on February 11, 2015. All participants in the community readiness interviews, the PA intervention, and the qualitative interviews receive written and oral information about the study. All interviewees give informed consent for their data to be used.

## Discussion

PA can be conceptualized as a key health resource for an ageing community. Enhanced PA reduces risks for many chronic diseases and injuries. In addition, physically active persons play more active roles in their respective communities than those engaging in less PA [29]. However, more evidence on how to effectively achieve these combined benefits of PA is needed. The community readiness concept goes beyond the individual level and applies a stage-based behaviour change model to the community level [18, 19] . The proposed AEQUIPA network builds on a regional approach both for developing, implementing and testing new behavioural and community-oriented interventions. Community capacity building approaches to reach and engage vulnerable groups and local stakeholders in their communities are increasingly being recognised as effective ways of reducing inequalities in access to services and interventions. They pave the way for sustainable program implementation in preventive research [15–17]. The *Ready To Change* study aims to improve our understanding of how community-based approaches to PA interventions for older adults may enhance programme reach. It will provide an estimate of the resources necessary to achieve equitable access to PA programs for vulnerable older adults, identify reasons for non-participation in PA interventions among older adults and provide information on how barriers to participation can be tackled.

The strength of the study is that it applies a structured approach to measure community resources and preparedness. Moreover, while strengthening community capacities is often recommended as an important strategy for equitable and sustainable health promotion, this is one of the few studies that aims to assess the effects of capacity building efforts in a controlled intervention study. A limitation of this study is the small number of municipalities in the study groups, which negatively affects our ability to control for differences between the included municipalities. Inclusion of more municipalities is unfortunately not possible due to budget restrictions. Another limitation is that we use reach as the primary outcome parameter ignoring other possible effects of capacity building. For example, Labonte et al. have argued that community capacity building is an aim in itself helping to cope with diverse problems in the community [30]. Furthermore, sustainability of the health promotion activities is assumed to be positively affected by community readiness and capacities. Regarding this aspect, it is planned to assess the readiness and the activities in the municipalities once again in a second phase of this study.

Despite these limitations, we expect that our research work will increase the knowledge on effective and targeted recruitment approaches and will help policy makers to invest resources in the most effective long-term PA efforts. In addition, it will provide a direction for health and behavioural scientists to explore further possibilities to enhance PA levels across population subgroups.

## Abbreviations

AEQUIPA: physical activity and health equity: primary prevention for healthy ageing
BMBF: federal ministry of education and research
ICER: incremental cost-effectiveness rate
PA: physical activity
RE-AIM: reach efficacy adoption implementation maintenance
RTC: ready to change
WHO: world health organization
